# Spermatic Cord Hydrocele: A Case Series

**DOI:** 10.7759/cureus.102740

**Published:** 2026-01-31

**Authors:** Sukesh K S, Kiran Prabhakar Rebello, Anuj Jain, Deepika H G

**Affiliations:** 1 General Surgery, Autonomous State Medical College, Sultanpur, Sultanpur, IND; 2 Anesthesia, Autonomous State Medical College, Sultanpur, Sultanpur, IND

**Keywords:** encysted hydrocele, hydrocele, infant hydrocele, inguinoscrotal swelling, transilluminant

## Abstract

Although hydrocele is one of the most common inguinoscrotal pathologies, spermatic cord hydrocele (SCH) is a rare entity. Its development is attributed to the incomplete closure of the processus vaginalis, resulting in a collection of fluid that may either communicate with or become encysted along the spermatic cord. Despite its rarity, it has been documented in children and, more rarely, in adults. As it presents with an inguinoscrotal swelling separate from the testis, the encysted variant of SCH poses a diagnostic dilemma when differentiating it from an irreducible inguinal hernia; however, its nature of being transilluminant, along with a complete clinical examination, aids in diagnosis. This study, conducted at a peripheral tertiary care center in northern India, aimed to describe the clinical presentation, diagnostic challenges, and surgical management of SCHs across different age groups, highlighting the importance of thorough clinical examination and ultrasonography in distinguishing this entity from inguinal hernia. We report six cases - two adult patients and four pediatric patients - with SCH, including five with the encysted variant and one with the communicating type, all of whom were managed surgically at our institute. The inclusion of both pediatric and adult cases makes this case series unique.

## Introduction

Hydrocele is the accumulation of excess fluid between the parietal layer and the visceral layer of the tunica vaginalis covering the testis, and it is one of the most commonly encountered scrotal pathologies. Spermatic cord hydrocele (SCH), on the other hand, is a rare condition usually seen in infants and children, and more rarely in adults, due to the incomplete closure of the processus vaginalis or a patent processus vaginalis [1,2]. SCH is classified into two types: one that communicates with the peritoneal cavity, termed a communicating hydrocele, and another that lacks such communication, termed an encysted hydrocele. The global prevalence of hydrocele in infants and children is estimated at one per 1000 and 3.4 per 1000, respectively [3]. SCH constitutes 5.4% of these cases, but it is extremely rare in adults [4]. The presentation as an irreducible inguinoscrotal swelling usually poses a diagnostic dilemma with an irreducible or incarcerated inguinal hernia [4].

In the present study, we report six cases of SCH encountered over a period of two years, managed surgically at a peripheral tertiary care center in northern India. The objective was to describe the clinical presentation, diagnostic challenges, and surgical management of SCHs across different age groups and to highlight the importance of thorough clinical examination and ultrasonography in distinguishing this entity from an inguinal hernia.

## Materials and methods

This is a retrospective case series of patients diagnosed with SCH and managed at the Department of General Surgery, Autonomous State Medical College, Sultanpur, Uttar Pradesh, India, a peripheral tertiary care institute in northern India. After obtaining written informed consent, all patients with a preoperative or intraoperative diagnosis of SCH were enrolled in this study over a period of two years from December 2023 to December 2025.

Patient demographic data, clinical presentation, blood investigations, intraoperative findings, treatment, outcomes, and complications were studied. Patients who were unfit for surgery, did not consent to the study, or had other scrotal pathologies, as well as those with discrepancies in pre- and postoperative diagnosis, were excluded. The diagnosis of SCH was made through detailed clinical examination and ultrasound. The diagnosis was confirmed based on intraoperative findings and formal histopathological examination performed by experienced pathologists.

Each case has been described with relevant history, examination findings, diagnostic challenges, intraoperative findings, surgical management, and postoperative outcomes.

## Results

Case presentations

Case 1

**Figure 1:**
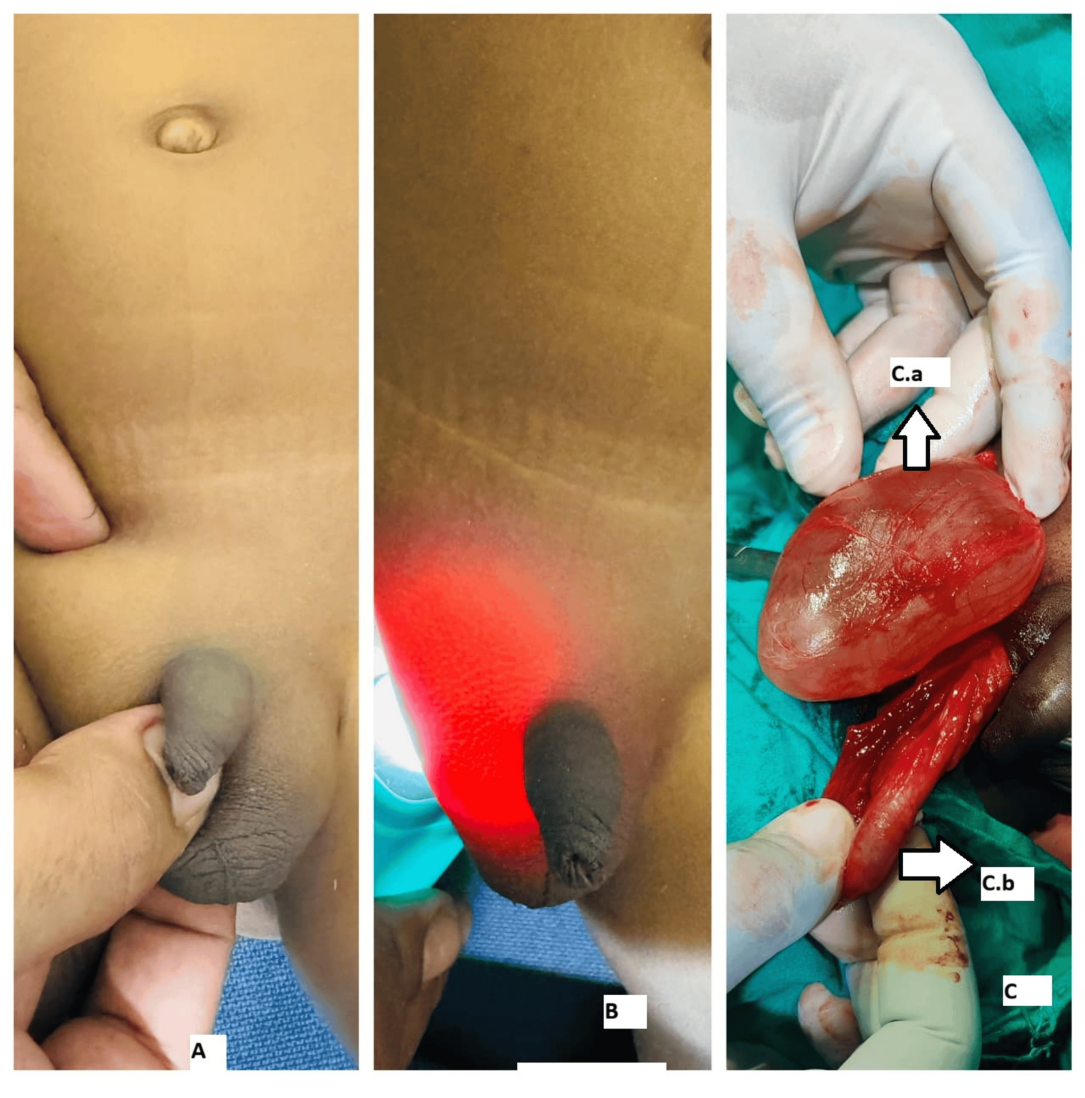
Examination and intraoperative findings of case 1 (A) Irreducible swelling of the right hemiscrotum. (B) Transilluminant swelling of the right hemiscrotum. (C) Intraoperative image showing an encysted hydrocele. C.a, Encysted hydrocele; C.b, Testis and cord.

A three-year-old male patient presented with right scrotal swelling for one year, which had progressively increased in size and showed no change in size with position or crying. Clinical examination revealed a 4 x 3 cm swelling in the right hemiscrotum (Figure 1A) that was firm, irreducible, and transilluminant (Figure 1B). The testis and cord above the swelling were separately palpable. A provisional diagnosis of SCH was made, and ultrasonography confirmed the diagnosis. Under caudal block with sedation, a scrotal exploration and excision of the SCH (Figure 1C) were performed. The postoperative period was uneventful. Histopathological analysis confirmed the diagnosis of SCH. On follow-up, the patient was doing well and relieved of symptoms.

Case 2

**Figure 2:**
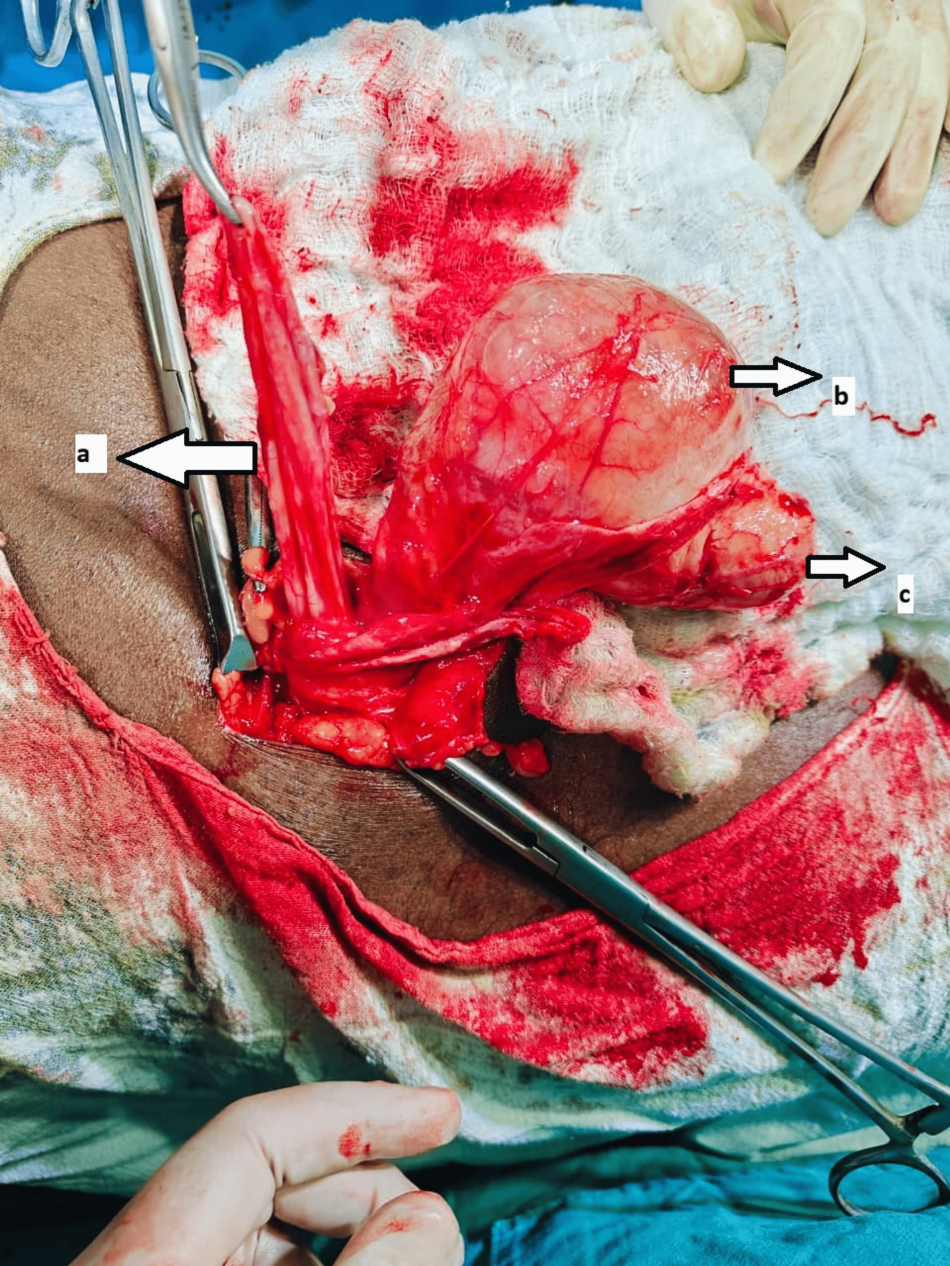
Intraoperative image of case 2 showing encysted hydrocele with direct inguinal hernia a, Sac of direct inguinal hernia; b, Encysted hydrocele; c, Testis.

A 62-year-old male patient, agriculturist by profession with no comorbidities, presented with a right inguinoscrotal swelling for three years, which had progressively increased in size and showed no change in size with position or physical activities. Clinical examination revealed a 5 x 3 cm swelling in the right hemiscrotum that was firm, irreducible, and transilluminant. The testis and cord above the swelling were separately palpable, with a reducible 3 x 2 cm swelling medial to the superficial ring of the inguinal canal showing a positive cough impulse. A provisional diagnosis of SCH with a direct inguinal hernia was made, and ultrasonography confirmed the diagnosis. Under spinal anesthesia, through inguinal exploration (Figure 2), the diagnosis of SCH with direct inguinal hernia was confirmed, and excision of the SCH with Lichtenstein’s hernioplasty was performed. The postoperative period was uneventful. Histopathological analysis confirmed the diagnosis of SCH. On follow-up, the patient was doing well and relieved of symptoms.

Case 3

**Figure 3:**
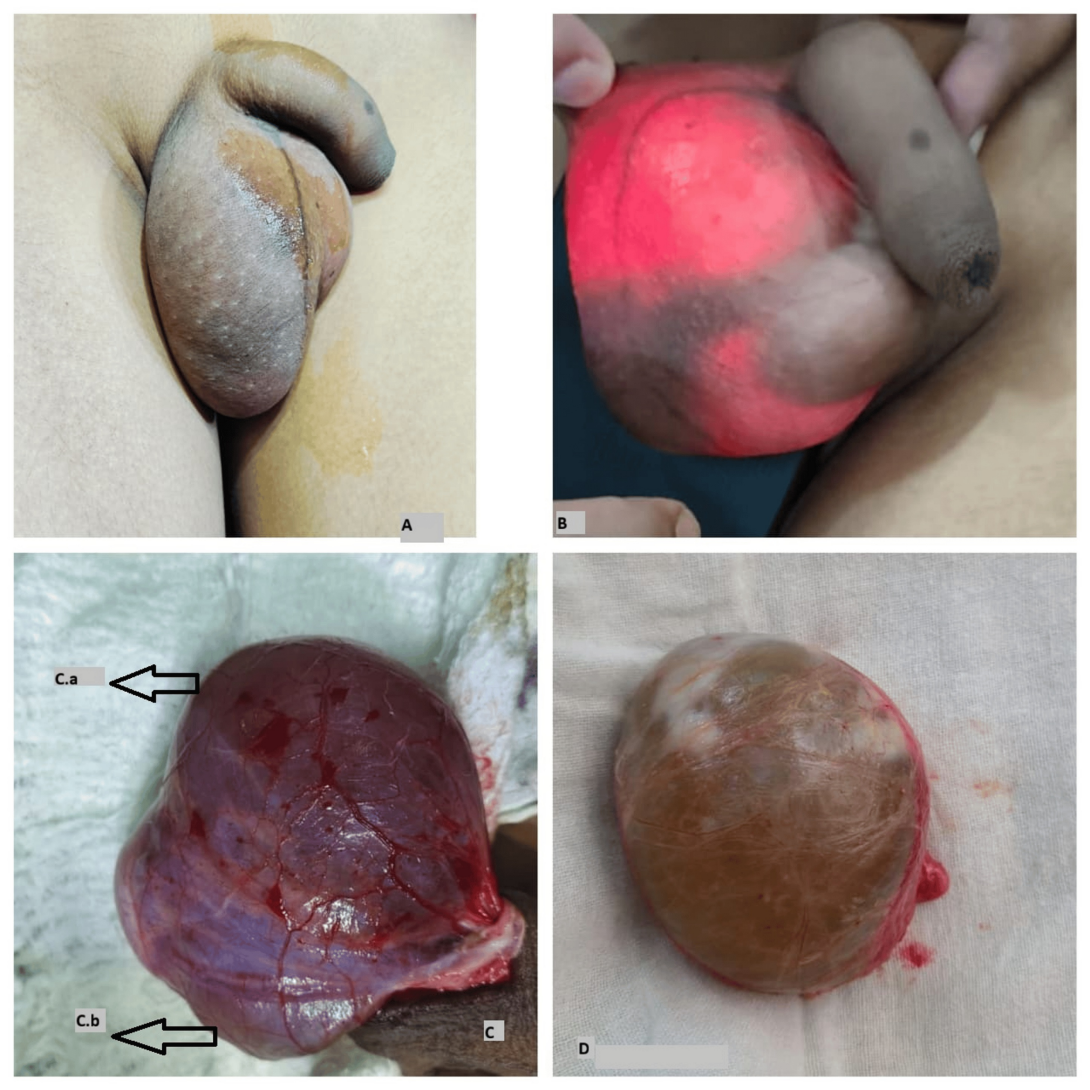
Clinical and intraoperative images of case 3 (A) Irreducible inguinoscrotal swelling. (B) Transilluminant inguinoscrotal swelling. (C) Intraoperative view. C.a, Testis; C.b, Encysted hydrocele. (D) Specimen of encysted hydrocele.

A 14-year-old male patient presented with right scrotal swelling for three years, which had progressively increased in size and showed no change in size with position or exertion. Clinical examination revealed a 5 x 4 cm swelling in the right hemiscrotum that was firm, irreducible (Figure 3A), and transilluminant (Figure 3B). The testis and cord above the swelling were separately palpable. A provisional diagnosis of SCH was made, and ultrasonography confirmed the diagnosis. Under spinal anesthesia, through scrotal exploration, excision of the right SCH (Figures 3C-3D) was performed. The postoperative period was uneventful. Histopathological analysis confirmed the diagnosis of SCH. On follow-up, the patient was doing well and relieved of symptoms.

Case 4

**Figure 4:**
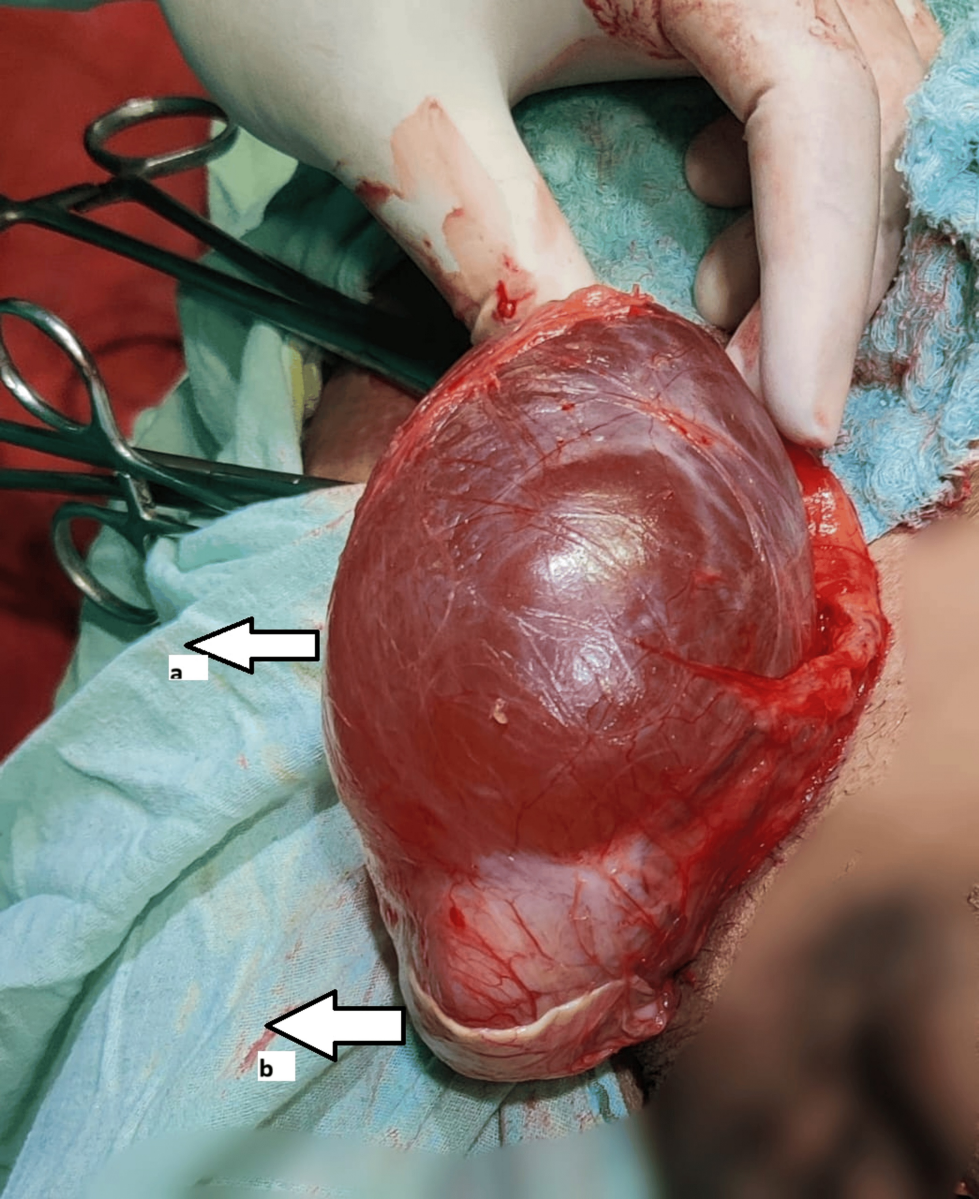
Intraoperative image of case 4 a, Encysted hydrocele; b, Testis.

A 42-year-old male patient, shopkeeper by profession with no comorbidities, presented with an inguinoscrotal swelling for three years, which had progressively increased in size and showed no change in size with position or physical activities. Clinical examination revealed a 6 x 3 cm swelling in the right hemiscrotum that was firm and irreducible. This history and examination suggested a diagnosis of irreducible inguinal hernia; however, the swelling was transilluminant, and the testis and cord above the swelling were separately palpable. A provisional diagnosis of SCH was made, and ultrasonography confirmed the diagnosis. Under spinal anesthesia, excision of the SCH (Figure 4) was performed. The postoperative period was uneventful. Histopathological analysis confirmed the diagnosis of SCH. On follow-up, the patient was doing well and relieved of symptoms.

Case 5

**Figure 5:**
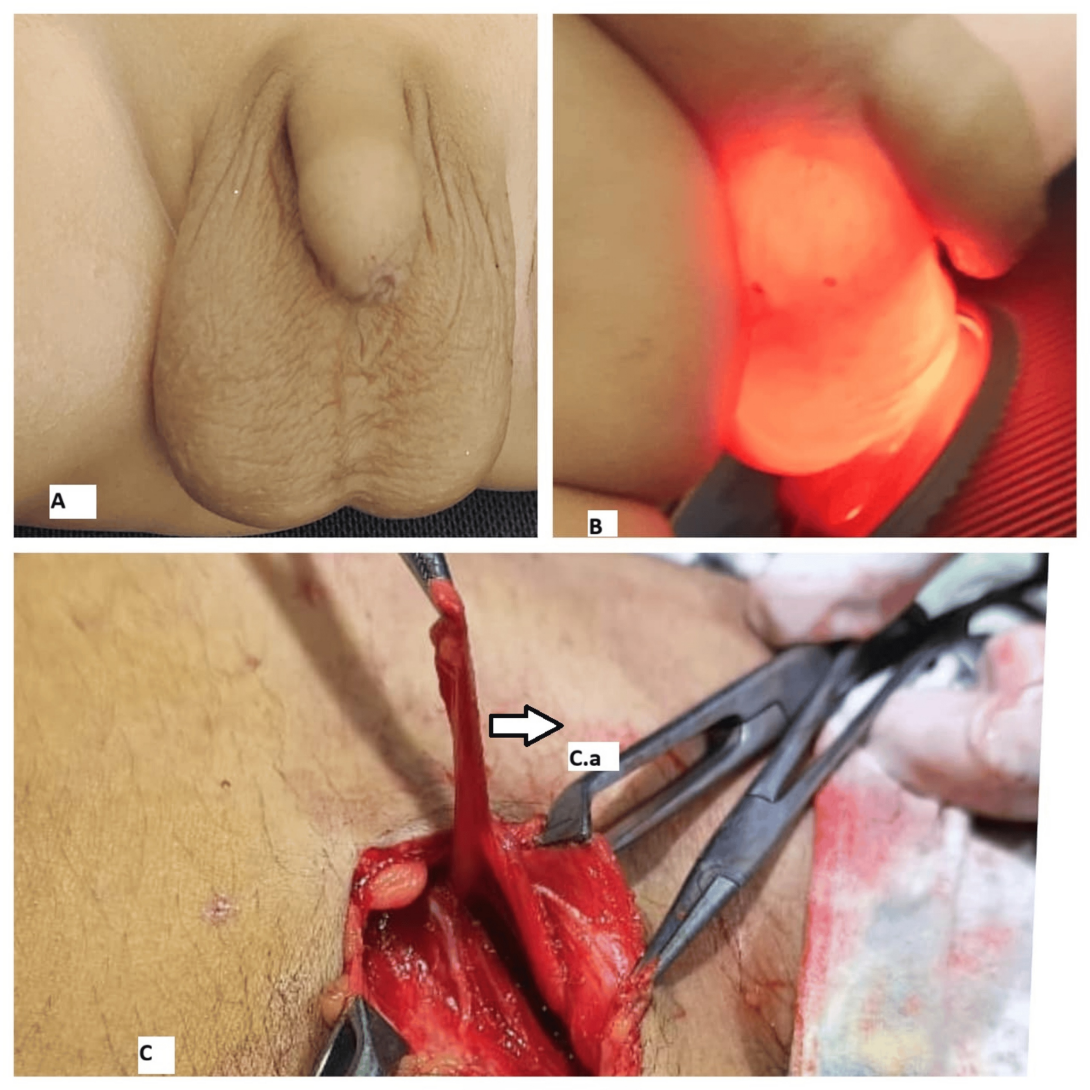
Clinical and intraoperative images of case 5 (A) Reducible swelling of the right hemiscrotum. (B) Transilluminant swelling of the right hemiscrotum. (C) Intraoperative view. C.a, Communicating patent processus vaginalis.

A four-year-old male patient presented with right scrotal swelling for one year, which had progressively increased in size and was relieved when lying down but increased during exertion. Clinical examination revealed a 4 x 3 cm swelling in the right hemiscrotum (Figure 5A) that was soft, reducible, and transilluminant (Figure 5B). The testis was separately palpable. A provisional diagnosis of SCH (communicating type) was made, and ultrasonography confirmed the diagnosis, showing anechoic compressible swelling arising from the inguinal canal to the base of the testis with no intra-abdominal contents in the standing and Valsalva positions. Under caudal block with sedation, through inguinal exploration, ligation of the remnant processus vaginalis (Figure 5C) was performed. The postoperative period was uneventful. Histopathological analysis confirmed the diagnosis of SCH. On follow-up, the patient was doing well and relieved of symptoms.

Case 6

**Figure 6:**
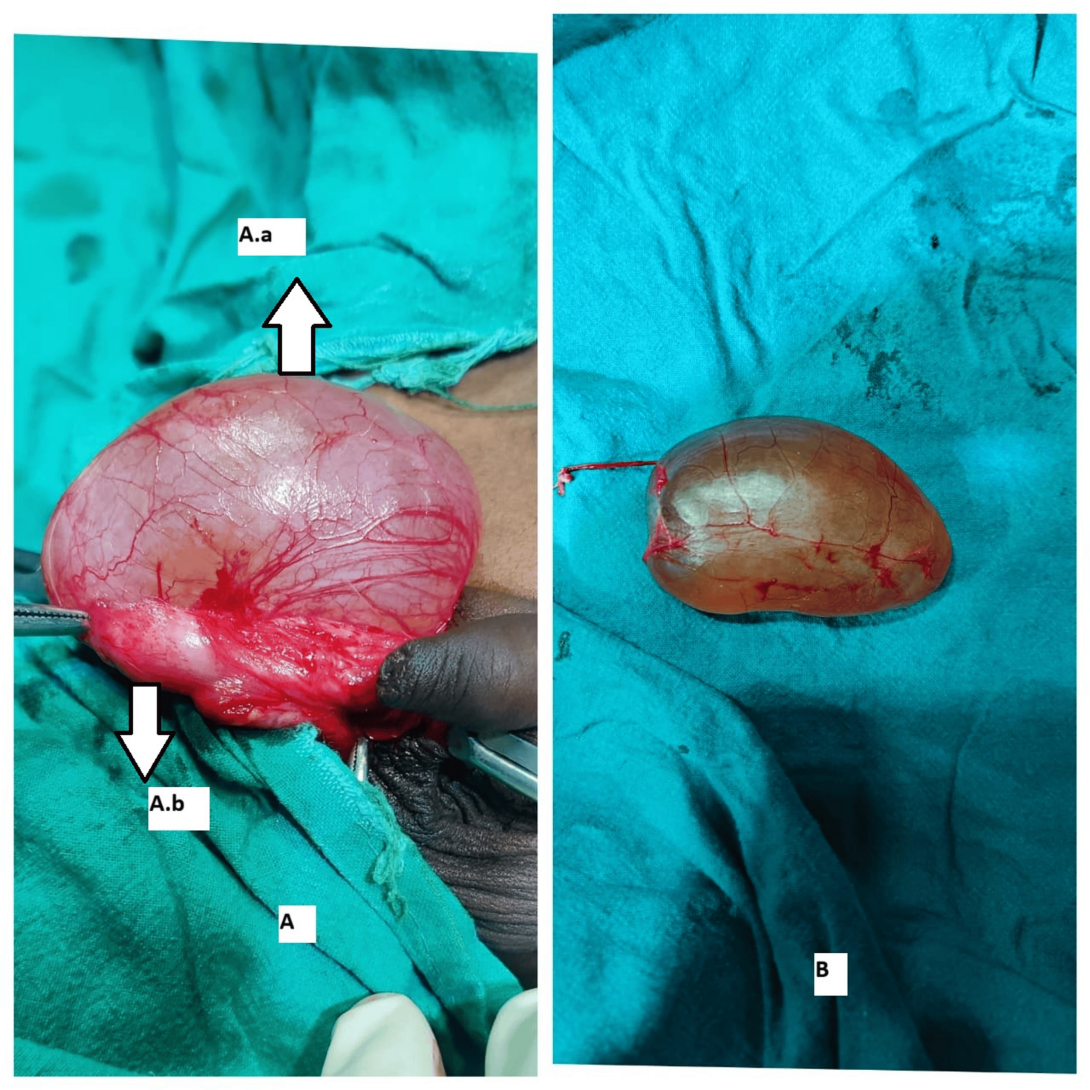
Intraoperative images of case 6 (A) Intraoperative view. A.a, Testis; A.b, Encysted hydrocele. (B) Specimen of encysted hydrocele.

A 10-year-old male patient presented with right scrotal swelling for two years, which had progressively increased in size and showed no change in size with position or exertion. Clinical examination revealed a 4 x 3 cm swelling in the right hemiscrotum that was firm, irreducible, and transilluminant. The testis and cord above the swelling were separately palpable. A provisional diagnosis of SCH was made, and ultrasonography confirmed the diagnosis. Under spinal anesthesia, through scrotal exploration, excision of the SCH (Figure 6) was performed. The postoperative period was uneventful. Histopathological analysis confirmed the diagnosis of SCH. On follow-up, the patient was doing well and relieved of symptoms.

Summary of cases

Key details of all six cases are presented in Table 1.

**Table 1:** Clinical and surgical details of the six cases with SCH SCH: Spermatic cord hydrocele

Case	Age (years)	Symptoms	Examination	Diagnosis	Management
1	3	Right scrotal swelling	4 x 3 cm swelling in the right hemi-scrotum, which was irreducible, transilluminant, and separate from the testis.	Right SCH	Excision of SCH through scrotal exploration under caudal block with sedation.
2	62	Right inguinoscrotal swelling for three years	5 x 3 cm swelling in the right hemi-scrotum, which was firm, irreducible, and transilluminant. Testis and cords above the swelling were palpable, with a reducible 3 x 2 cm swelling medial to the superficial ring of the inguinal canal showing positive cough impulse.	Right SCH with right direct inguinal hernia	Excision of the SCH with Lichtenstein’s hernioplasty through the inguinal approach under spinal anesthesia.
3	14	Right scrotal swelling for three years	5 x 4 cm swelling in the right hemi-scrotum, which was irreducible, transilluminant, and separate from the testis.	Right SCH	Excision of SCH through scrotal exploration under spinal anesthesia.
4	42	Right inguinoscrotal swelling for three years	6 x 3 cm swelling in the right hemi-scrotum, which was irreducible, transilluminant, and separate from the testis.	Right SCH	Excision of SCH through scrotal exploration under spinal anesthesia.
5	4	Right scrotal swelling for one year	4 x 3 cm swelling in the right hemi-scrotum, which was soft, reducible, but transilluminant. The testis was separately palpable.	Right SCH (communicating type/funicular hydrocele)	Inguinal exploration with ligation of the communicating tract (similar to herniotomy) under caudal block with sedation.
6	10	Right scrotal swelling for two years	4 x 3 cm swelling in the right hemi-scrotum, which was firm, irreducible, and transilluminant.	Right SCH	Excision of SCH through scrotal exploration under spinal anesthesia.

## Discussion

During embryogenesis, between the 28th and 32nd weeks of gestation, the testes descend from their initial intra-abdominal location into the scrotum, carrying two layers of the peritoneum with them. This structure is called the processus vaginalis, which atrophies over time. After atrophy, the vaginal process leaves a thin membrane surrounding the testes and epididymis called the tunica vaginalis [5,6]. Failure of this atrophy is referred to as patent processus vaginalis, and the extent of patency can give rise to various pathologies. If the abdominal contents communicate into the scrotum via the patent processus vaginalis, it is termed a hernia, and if it contains only fluid, it is called a hydrocele [1,5].

Three main types of cord hydroceles have been described based on the extent of closure of the processus vaginalis: continuous, funicular, and encysted. When there is a failure of complete closure of the vaginal process, it allows fluid to flow freely between the abdominal cavity and the tunica vaginalis, resulting in a continuous hydrocele. If the closure fails distally while the proximal end remains intact, it results in a connection between the tunica vaginalis and the abdominal cavity through a narrow passage, known as a funicular hydrocele. If a section of the vaginal process (Cranford's section) between the distal space above the epididymis and testis up to the internal inguinal ring fails to close, it results in a fluid-filled sac within the scrotum, which does not communicate with the abdominal cavity, referred to as an encysted hydrocele [5,6].

Cord hydrocele is usually common in neonates and typically resolves within a year. Although rare in children, a study found cord hydrocele in 1.5% of children evaluated for inguinal swelling [7]. It is extremely rare in adults [1-4,7].

Clinically, cord hydrocele presents as a painless inguinoscrotal/inguinal/scrotal swelling. A detailed clinical examination is essential to differentiate it from other causes, such as inguinal hernia, epididymal cyst, spermatic cord lipoma, testicular tumor, scrotal edema, and varicocele. An encysted hydrocele presents as an irreducible swelling in the scrotum, often extending to the inguinal region if large, which may create a dilemma with an incarcerated/irreducible inguinal hernia, but its characteristic brilliant transillumination confirms the diagnosis. In contrast, the communicating type or funicular hydrocele presents as a reducible swelling that is also transilluminant [6,7-10]. Cases 1, 2, 3, 4, and 6 were of the encysted type, while case 5 was of the communicating type.

Ultrasonography helps confirm the diagnosis by showing an anechoic mass in the scrotum, which is avascular, sharply demarcated, and separate from the testes and epididymis, with or without septations [8]. Management of an encysted hydrocele typically involves excision, whereas the communicating type is managed with obliteration of the tract, similar to the principles of hernia. Cases 1, 2, 3, 4, and 6 were managed by excision, and for case 5, herniotomy was performed. Case 2 had a direct hernia along with SCH, which was managed by hernioplasty with excision [6-9]. 

Patients generally have a good prognosis after operative intervention, with very low rates of recurrence. Usual complications may include subcutaneous hematoma, serous secretion from the wound, seroma formation, surgical site infection, swelling of the scrotum, chronic pain, and wound dehiscence [10]. None of our patients experienced any postoperative complications. 

Histopathological examination of the excised specimens typically reveals a thin layer of dense collagenous tissue containing elastic fibers with smooth muscle bundles lined by mesothelium [8]. All cases in our series demonstrated a similar histological picture, confirming the diagnosis.

## Conclusions

SCH is a rarely encountered entity among all inguinoscrotal swellings in clinical practice. There are occasions when it is confused with an irreducible inguinal hernia, particularly in adults, and only during exploration do the findings differentiate the two. A detailed clinical examination helps to distinguish it from an irreducible inguinal hernia, while ultrasonography aids in confirmation. Surgical management offers a good prognosis with fewer complications. This study presents a series of cases encountered over a period of two years in a peripheral tertiary center in northern India, involving both pediatric and adult patients, and highlights the importance of clinical evaluation to avoid emergency intervention.
